# Epidemiological and some clinical characteristics of neuroblastoma in Mexican children (1996–2005)

**DOI:** 10.1186/1471-2407-9-266

**Published:** 2009-08-03

**Authors:** Servando Juárez-Ocaña, Virginia Palma-Padilla, Guadalupe González-Miranda, Alicia Georgina Siordia-Reyes, Enrique López-Aguilar, Martha Aguilar-Martínez, Juan Manuel Mejía-Aranguré, Rogelio Carreón-Cruz, Mario Enrique Rendón-Macías, Arturo Fajardo-Gutiérrez

**Affiliations:** 1 Unidad de Investigación Médica en Epidemiología Clínica, Hospital de Pediatría, Centro Médico Nacional Siglo XXI, Instituto Mexicano del Seguro Social, Ciudad de México, México; 2 Servicio de Patología, Hospital de Pediatría, Centro Médico Nacional Siglo XXI, Instituto Mexicano del Seguro Social, Ciudad de México, México; 3 Servicio de Oncología, Hospital de Pediatría, Centro Médico Nacional Siglo XXI, Instituto Mexicano del Seguro Social, Ciudad de México, México; 4 Servicio de Oncología Pediátrica, Hospital General, Centro Médico La Raza, Instituto Mexicano del Seguro Social, Ciudad de México, México

## Abstract

**Background:**

Neuroblastoma (NB) is the principal tumor of the sympathetic nervous system in children under one year of age. The incidence in developed countries is greater than that in developing countries. The aim of this article is to present the epidemiological and some clinical characteristics of Mexican children with NB.

**Methods:**

A population-based, prolective study, with data obtained from the Childhood Cancer Registry of the Instituto Mexicano de Seguro Social. Statistical analysis: The simple frequencies of the variables of the study and the annual average incidence (per 1,000,000 children/years) by age and sex were obtained. The trend was evaluated by calculating the annual percentage of change. The curves of Kaplan-Meyer were employed for the survival rate and the log-rank test was used to compare the curves.

**Results:**

Of a total of 2,758 children with cancer registered during the period from 1996–2005, 72 (2.6%) were identified as having Group IV, defined according to the International Classification of Childhood Cancer. The incidence for NB was 3.8 per 1,000,000 children/year; NB was highest in the group of children under one year of age, followed by the group of children between the ages 1–4 years (18.5 and 5.4 per 1,000,000 children/years, respectively). The male/female ratio was 1.1 and there was no trend toward an increase. The time of diagnosis was 26 days (median), but varied according to the stage at diagnosis. Stages III and IV were presented in 88% of the cases. There was no association between the stage, the age at time of diagnosis, or the histological pattern. The overall five-year survival rate was 64%; the patients with stage I, II, III, or IVs did not die; and the five-year survival rate of cases in Stage IV was 40%.

**Conclusion:**

It is possible that the low incidence of neuroblastoma in Mexican children is due to the difficulty in diagnosing the cases with the best prognosis, some of which could have had spontaneous regression. There was no trend to an increase; the majority of the cases were diagnosed in the advanced stages; and the overall five-years survival rate was similar to that for developed countries.

## Background

During fetal life, cells of the neural crest colonize the sympathetic ganglions, the medulla of adrenal glands, and other sites. Neuroblastoma (NB) and related tumors [ganglioneuroblastoma (GNB) and anaplastic ganglioglioma] arise from these cells. These neoplasias are in the family of tumors characterized by diverse biological and clinical behaviors that range from spontaneous regression or differentiation into benign neoplasias, principally in children under one year of age, to very aggressive metastasis in older children [1,2]

According to the International Classification of Childhood Cancer (ICCC) [3], NB and other tumors of the peripheral nerve cells (NB-O) are classified in Group IV. Together, NB and GNB comprise 97% of the tumors in this group [4]. The adrenal gland is the topographical site where NB is most frequently found [1].

NB represents between 8–10% of the total cancers in children (0–14 years) in countries such as the United Stated of America (USA), Australia, and those in Europe, whereas the frequency is generally lower (3%) in Latin-American countries and in some cities in Asia, such as Tianjin, China, and Delhi and Madras, India [5]. In developed countries, the annual average incidence of NB in children varies between 7 and 14 per 1,000,000 children/years; in some of these countries, NB occupies fourth place in incidence. In developing countries, however, the incidence is low, generally having been reported to be less than 6 per 1,000,000 children/year [5].

We have reported that, in Mexico, the frequency and incidence of NB (2.7% and 3.6 per 1,000,000/year, respectively) are similar to the values found for developing countries [6]; however, in that study, we reported neither the trends, nor the incidence according to age, sex, or clinical characteristics, such as the time of diagnosis (TD), the stage, and the five-year survival rate of the patients. For this reason, the objective of this article is to present the clinical and epidemiological characteristics of NB in the cases studied.

## Methods

### Type of study

Population-based, prolective study.

### Study population

New cases of NB and NB-O in children under 15 years of age, who fulfilled the following criteria: they had the right to service from IMSS; were attended either in the Pediatric Hospital (PH) of the Siglo XXI National Medical Center of IMSS or in the Pediatric Oncological Service of the General Hospital La Raza Medical Center, IMSS; and were residents either in Mexico City or in the following four states of the Republic of Mexico: Mexico State, Morelos, Guerrero, or Chiapas.

### Study period

From January 1, 1996 to December 31, 2005.

### Process for collection of data

The data were obtained from the Registry of Childhood Cancer of the Instituto Mexicano de Seguro Social (RCN-IMSS) that is maintained in the Research Unit of Clinical Epidemiology of the PH. This Registry was started in 1996 and is ongoing. IMSS is the principal institution that provides medical attention in Mexico, serving 41% of the population of the country. The method of collection and the coding and registry of data in the RCN-IMSS have been described in detail [6]. Therefore, in this paper, we will summarize those aspects that are most relevant for the registry of cases of NB and NB-O.

All the cases of NB and NB-O, which were registered during the period of study, were analyzed. The cases were coded following their morphology and topography, in accord with the third edition of the International Classification of Disease for Oncology [7]; in all cases, the diagnosis was confirmed by histopathological studies. The site of origin of the tumor in the child's body (topography) was determined from the clinical data taken when the patient presented, from the imaging studies (radiographic, ultrasound, and/or tomography), and from the surgical findings when the child underwent surgery. Also, to evaluate the metastasis of the tumor at the TD when surgery was used either as a method of diagnosis and/or treatment, the criteria of the International Neuroblastoma Staging System [8] were used. In those cases in which surgery was not performed, the criteria of the Children's Cancer Group Neuroblastoma Staging system [9] were used. Also, the prognostic evaluation of the patients with NB (favorable or unfavorable histology) was carried out by using the criteria of the International Neuroblastoma Pathology Classification System [10]. This last technique was used in only 22 cases, because this diagnostic tool was performed in only one hospital (PH) included in the RCN-IMSS.

The family history of cancer was obtained through the analysis of the genealogical trees of each patient with NB for the 1^st^, 2^nd^, and 3^rd ^generation (siblings, parents, aunts, uncles and grandparents). The survivor rate for NB was determined though a five-year follow-up of the patients (localization of the patients was done by direct contact at the hospital or at home, from medical records, institutional archives, telephone, and/or mail).

The TD (also known as lag time) is defined as the period between the start of the symptoms and the date of the histopathological diagnosis of NB; the lag time was registered in days [11].

To evaluate the internal consistency in the collection of data and the capture of same, the program Child Check was used to detect and correct any inconsistencies [12].

### Denominators

The reference populations (denominators) were obtained from the structure of the population assigned to a family physician of the IMSS [13].

### Analysis

The simple frequencies of the different types of cancer, following Group IV of the ICCC, and the topography, stage, grade, histological differentiation of Shimada [10], and family history were obtained. Also, the annual average incidence per period (AAIP; per 1,000,000 children/year) was estimated. The AAIP was standardized by age through the direct method, with the value for the world population taken as reference population [14]. AAIP was stratified by age and sex. The trends of the incidence during the study period was evaluated by calculating the average annual percent change (AAPC) and the confidence interval at 95% (CI_95%_) [15]. For the TD, the median, and interquartile ranges (25–75^th ^percentile) were obtained. The association between the stage and the median TD was analyzed by using Kuskal-Wallis statistics. The Kaplan-Meyer method was used for survival rate analysis and the Log-rank test was used to compare the curves [16]

## Results

During the period under study, there was a total of 2,758 new cases of childhood cancer, of which 72 (2.6%) were determined to be Group IV of the ICCC. The frequency of the subgroup of NB was 94.4% (Table 1).

**Table 1:** Frequency and incidence* of neuroblastoma and other peripheral nervous cell tumors in Mexican children (1996–2005)

Diagnostic group	n	%	ASR**
**IVa. Neuroblastoma and Ganglioneuroblastoma**	**68**	**94.4**	**3.80**
*- Neuroblastoma*	*58*	*80.5*	*3.30*
*- Ganglioneuroblastoma*	*10*	*13.9*	*0.50*
			
**IVb. Other peripheral nervous cell tumors**	**4**	**5.6**	**0.16**
*- Medulloepithelioma*	*1*	*1.4*	*0.04*
*- Neuroepithelioma*	*1*	*1.4*	*0.04*
*- Olfatory Neuroblastoma*	*2*	*2.8*	*0.08*
**TOTAL**	**72**	**100.0**	**3.96**

*Rate per 1,000,000 children/year; **ASR: Age-standardized rates

The incidence of NB for the period under study was 3.8 cases per 1,000,000 of children/year and the male/female ratio was 1.1 (Table 2). According to age, the highest incidence was found in children under one year of age and the second highest was in the 1–4 year-old age group, the rates being 18.5 and 5.4 cases per 1,000,000 children/year, respectively. In the groups 5–9 year-olds and 10–14 year-olds, the incidence was very low. Median age at diagnosis was 27 months [interquartile range (25–75^th^) 8.5 and 48 months]. During the period under study, the trends of NB remained stable (AAPC = 2.3; CI_95%_, -8.7, +14.5) (Table 3).

**Table 2:** Incidence* of neuroblastoma in Mexican children by age and sex (1996–2005)

				Sex	
				
	Overall		Male		Female		M/F	
**Age Group** **(years)**	**n**	**rate**	**n**	**rate**	**n**	**rate**	**Ratio**

**< 1**	26	18.5	16	26.9	10	17.6	1.6
**1 – 4**	32	5.4	14	4.6	18	6.1	0.8
**5 – 9**	8	1.1	3	0.8	5	1.3	0.6
**10 – 14**	2	0.2	2	0.6	0	0.0	-
**AAIP**	68	2.9	35	3.1	33	3.1	1.0
**ASR**	68	3.8	35	3.9	33	3.7	1.1

*Rates per 1,000,000 children/year; n, number of cases; M, Male; F, Female; AAIP, Average Annual Incidence per Period; ASR, Average Standardized Rate.

**Table 3:** Trend* of neuroblastoma in Mexican children (1996–2005)

Year	1996	1997	1998	1999	2000	2001	2002	2003	2004	2005		
			
	n = 9	n = 3	n = 6	n = 7	n = 4	n = 6	n = 12	n = 8	n = 8	n = 5	AAPC	**CI**_95%_
**ASR**	5.6	1.8	3.3	3.9	2.0	3.0	6.8	4.7	4.3	2.8	2.3	-8.7,+14.5

*Rates per 1,000,000 children/year; n, number of cases; AAPC, Average Annual Percentage Change; CI_95%_, Confidence Interval; ASR, Average Standardized Rate (0 – 14 years).

As for topography, 63.9% of the tumors were in the abdominal area (34.5% in the adrenal gland), 12.1% in the face or neck, and 10.3% in the thorax and 10.3% in the pelvis; the site of origin could not be determined in only 3.4% of the cases. Of the 68 cases with NB, 50 were staged. Of these 50, 88.0% were classified as Stage III, IV, or IVs. For children <1 year old, only two cases (11.1%) were localized stages (I and II) and 12 (66.7%) were disseminated stages (III and IV), with only four cases (22.2%) being Stage IVs (Table 4).

**Table 4:** Stage at diagnosis in Mexican children with neuroblastoma by age groups (1995–2006)

Age groups (years)	Stage at diagnosis											
	
	I		II		III		IV		IVs		Total	
	
	n	%	n	%	n	%	n	%	n	%	n	%
**< 1**	2	11.1	0	0.0	5	27.8	7	38.9	4	22.2	**18**	**100.0**
**1 – 4**	1	4.0	2	8.0	3	12.0	19	76.0	0	0.0	**25**	**100.0**
**5 – 9**	0	0.0	0	0.0	2	33.3	4	66.7	0	0.0	**6**	**100.0**
**10–14**	0	0.0	1	100.0	0	0.0	0	0.0	0	0.0	**1**	**100.0**
**Total**	**3**	**6.0**	**3**	**6.0**	**10**	**20.0**	**30**	**60.0**	**4**	**8.0**	**50**	**100.0**

n: Number of cases

The median TD was 26 days. The TD differed according to the stage (P = 0.01): the longest was found for Stage IV and the shortest for Stage I (median 35.5 and 3 days, respectively) (Table 5).

**Table 5:** Stage at diagnosis by time of diagnosis (*lag time) *in Mexican children with neuroblastoma (1996–2005)

Stage at diagnosis	n	Time of diagnosis* Md (25–75%)
**I**	3	3 (1 – 17)
**II**	3	26 (12 – 134)
**III**	10	11 (5 – 40)
**IV**	30	35.5 (15 – 47)
**IVs**	4	4 (1.5 – 6.5)
**Total**	**50**	26 (8 – 42)**

*Kruskal-Wallis = 12.6; df = 4; P = 0.01; n, number of cases; Md, Overall median and interquartile range (25–75^th ^percentile) in days.

Of the total number of cases, 27.6% had a family history of cancer. For the 22 cases in which the grade and histological differentiation of Shimada (favorable or unfavorable histology) were determined, no relation with the stage of diagnosis was found (Table 6).

**Table 6:** Stage at diagnosis by histology in Mexican children with neuroblastoma (1996–2005)

	Histology		
	
	Favorable	Unfavorable	Total*
	
Stage at diagnosis	n	n	N
**I**	0	1	1
**II**	0	0	0
**III**	0	2	2
**IV**	6	11	17
**IVs**	2	0	2
**Total**	**8**	**14**	**22**

*Fisher exact test (two tail) = 1.0 (Stage at diagnosis vs histology, two by two table)

**Figure 1:**
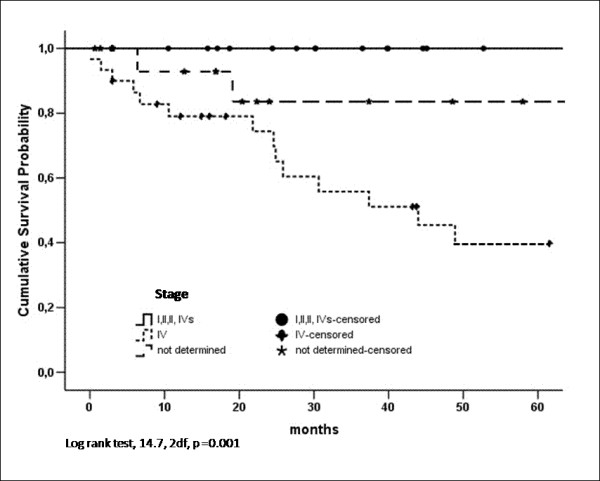
**Cumulative survival probabilitiy by stage for Mexican children with neuroblastoma (1996–2005)**.

The overall five-year survival rate was 64%. Only in those cases in Stage IV and those for which we could not determine the stage did the patient die, with the five-year survival rate for these cases being 85% and 40%, respectively (Figure 1).

## Discussion

In this study, we corroborated the finding that the incidence of NB in Mexican children is low in comparison to that reported for developed countries. In a previous study carried out in a population residing in Mexico City and having right to service from IMSS, an incidence of 3.2 cases per 1,000,000 children/year was found [17], a value that was thought to be an underestimation because the study was retrospective. However, this idea can be discarded because the incidence mentioned is very similar to the value (3.8 cases per 1,000,000 children/year) presented in the current, more robust study. In contrast to the prior study, the methodology used in the present study was of a much better quality: 1) the study was prolective; 2) the personnel that collected, coded, and captured the data had received better training in registering the cases of cancer; and 3) the diagnosis was confirmed be means of an histopathological testing in 100% of the cases.

The incidence reported here is similar to that for Latin American countries and to that for some Asian and African countries [5,18-20]. That is to say, it is probable that in underdeveloped countries the incidence of NB is truly much less than that reported for developed countries and that the difference is not due to incomplete registry of cases.

For this result, it is of interest to establish the cause(s) of this low incidence in the population of Mexican children studied. We hypothesize that some cases of NB in children under five years of age (an age group in which the incidence is higher) are not diagnosed and such cases undergo spontaneous regression without the presence of NB ever having been suspected in the patient, thus resulting in an lower incidence than that in developed countries.

In relation to the foregoing, it has been pointed out that NB can exist in three groups of patients: 1) a group, generally under one or two years of age, in which the tumor either disappears or matures into a benign tumor (ganglioneuroma) spontaneously or with minimum treatment; 2) a group, in which the tumor is curable with current therapeutics; and 3) a group in which NB is incurable with current therapy [21].

Mexico lacks the screening programs, such as those used in several developed countries, by which it has been shown that patients may have spontaneously regression in over 50% of the cases [22,23]. Due to the finding that, in those children less than one year of age, we obtained a much lower frequency in patients with NB in localized studies (11.1%) in comparison to that reported in developed countries (40.5 to 36.0%) [24-26], to the fact that, generally, the age at diagnosis was greater than two years old (median of 27 months), and to the fact that, in developed countries, these cases are diagnosed at a younger age (median 14.5 to 21.6 months) [27], we think that for Mexican children, and above all for those under five years of age, the phenomenon of spontaneous regression is presented. That is, the children develop the tumor and present non-specific symptoms; however, the doctors do not request a diagnostic study (such as an ultrasound in the case of abdominal symptoms) because seldom, within the limitations of the first level of treatment, do physicians suspect the presence of a neoplasm in a child [28]. Thus, the condition of the patient is left to evolve. If, in some cases, the tumor spontaneously regressed with the subsequent disappearance of symptoms, this would explain the lower incidence that we have encountered. We intend to corroborate this hypothesis in future studies. This hypothesis should be corroborated in future studies.

The above-mentioned scenario was reported by Powell *et al*. [27] in the late 1990's. These researchers found that the incidence of NB in the UK was lower (10.1 per 1,000,000 children/year) than that of other European countries (France, Germany, Austria, with 12.5, 11.4, 11.7 per 1,000,000 children/year, respectively). The age at diagnosis was greater than two years (median of 24.8 months), the frequency of the cases with localized tumors in those less than one year of age was lower in comparison to these countries and that of the cases with disseminated tumor (Stage IV) was greater in children over five years of age. The authors commented that their findings could be due to the different health-care systems in the various countries, but above all to less use of the technology (ultrasound) for the diagnosis of NB in the children of the UK. However, they did not mention whether the lower incidence could have been due to lack of diagnosis and the spontaneous regression in some cases of non-detected NB, such as we suspect happens in the population of Mexican children.

On the other hand, the incidence reported for Hispanic children residing in the United States is low in comparison to that for Caucasian children (8.5 vs. 11.5 cases per 1,000,000 children/year), but much higher then the value that we reported here (3.8 cases per 1,000,000 children/year) [29,30]. Therefore, we think that, in face of the lack of environmental factors known to be involved in the development of NB, it is highly probable that the higher incidence for Hispanic children in the U.S.A., in comparison to that of Mexican children, is due principally to an increased suspicion of the diagnosis and to the more frequent use of ultrasound technology for its diagnosis.

Also, we think that this possibility, the spontaneous regression of some NB, was what contributed to the results for the population in our study generally and, specifically, to that for the children under one year of age; the Stages III, IV, and IVs were the most frequent in 88.9% of the cases. This is consistent with the results of Spix *et al*. [2] who found, in children less than five years of age, an incidence higher than that which we reported. They mentioned that, because they did not know the stage of the cases that they analyzed, they did not find a higher frequency of localized cases in the youngest group. We also did not find such a situation; however, in our case, this was due to such cases not having been diagnosed. Thus, before considering the possibility that Mexican children may have a lower susceptibility and/or a lower exposure to risk factors for the development of NB, we must eliminate the possibility that this apparent lower susceptibility may only be due to reduced suspicion of NB and to the difficulty in establishing diagnosis of NB in such cases.

Although there was a trend to an increase similar to that reported by Six *et al*. for Eastern Europe (AAPC 2.8, P < 0.001) [2] and by Dalmasso *et al*. for the northwest of Italy (AAPC 2.3, CI_95% _1.0–2.5) [31], the trend that we found in the Mexican children was not statistically significant (AAPC 2.3, CI_95% _-8.7, +14.5). This result is possibly due to the short period (ten years) covered for our cases in the Registry. It will be necessary to evaluate the trend, in a more complete manner, over a longer period of registry.

In general, it has been found that the stages most frequently encountered at diagnosis are Stages III and IV (both of which are considered to be advanced stages) [32] or that 70% of the cases of children with NB present with metastasis at diagnosis [33,34]. Similarly, in another study carried out in Mexico, Stages III and IV were found in 80% of the cases [35]. The data in the present study were consistent with these results, in that the stages most frequently found were III and IV, with a frequency of 88% (Table 4). Due to this agreement between our data and those of various studies, we think that the registered cases included in the present study were cases without spontaneous remission.

With regard to the TD, our data (median of 26 days) for the population with NB were consistent with those reported in different studies that have found a median between 15 and 45 days [36-38]. However, the most important point is that the TD differed according to the stage of diagnosis, with Stage IV having the longest TD (median, 35.5 days). Nevertheless, we think that the association found between TD and the stage, although statistically significant, could be spurious because Stages I and II had only three cases each and because the longest TD was found in Stage II (the value of the 3^rd ^quartile was 134 days). Thus, we think that this association is not conclusive and that it will be necessary to increase the size of the sample for Stages I and II in order to have better supporting evidence.

With regard to the diagnostic stage and the histological differentiation of Shimada, the latter could only be performed in 32% of the cases (n = 22) and, similar to the literature, we did not find an association between a favorable histology and the localized stages (I and II), or between an unfavorable histological report and advanced stages (III and IV) [10]. Nevertheless, it will be necessary to perform a histological evaluation in all the cases that we study in the future in order to evaluate this correlation more precisely.

The survival rate found in our population of children with NB (overall five-year survival rate, 64%) was similar to that reported in the literature [2,15,33], and was a little higher than that reported in a prior study performed in Mexico (overall five-year survival, 53%) [35]. No patient with Stage I, II, III, or IVs died; for the children with Stage IV, the survival was 40%. These results indicated that the treatment offered to the patients with NB in our facilities was adequate. But only in 79.3% of the total number of cases did we have a complete follow-up of the patients. It is known that, when there are losses during the follow-up, the survival rate may be overestimated [39]. Because this is a possibility in our case, it will be very important to implement that will improve the follow-up of these patients.

Regarding genetic aspects, our results were consistent with those reported in the literature. It has been pointed out that NB occurs sporadically and that only for 2% or less of the patients is there a family medical history of NB; however, there is a great number of cases of distinct cancers in the family members of patients with NB [40]. In the present study, none of the 68 cases of NB had family histories of NB, but 27.6% of the cases did have a family history of other cancers. From these data, we think that there may exist a non-specific genetic susceptibility for NB.

## Conclusion

We conclude that the incidence of NB in Mexican children was low in comparison to that for children in developed countries, which possibly may be due to some cases, principally those of children under the age of five years, presenting spontaneous regression. There was no trend to an increase in the disease; 80% of the cases were diagnosed in advanced stages (III and IV), and the mean TD was 26 days. It will necessary to have an improved follow-up for patients with NB in order to bring about a better evaluation of the global survival.

## Competing interests

The authors declare that they have no competing interests.

## Authors' contributions

SJO recoded, analyzed the data, and wrote the first draft of the manuscript. VPP, GGM, RCC, ELA, MAM and MERM recoded, revised, and analyzed the database, and participated in the interpretation of results. AGSR performed the histopathological diagnosis, participated in the interpretation of results, and critically revised the manuscript. JMMA analyzed the data, participated in the interpretation of results, critically revised the manuscript, and provided guidance in some aspects of the project. AFG conceived and designed the study, analyzed the data, corrected the final manuscript, and provided guidance to all aspects of this project. All authors read and approved the final manuscript.

## Pre-publication history

The pre-publication history for this paper can be accessed here:

http://www.biomedcentral.com/1471-2407/9/266/prepub
